# Intercellular Transport of Oct4 in Mammalian Cells: A Basic Principle to Expand a Stem Cell Niche?

**DOI:** 10.1371/journal.pone.0032287

**Published:** 2012-02-16

**Authors:** Hans J. Rolf, Sabine Niebert, Marcus Niebert, Lena Gaus, Henning Schliephake, K. Günter Wiese

**Affiliations:** 1 Research Group Experimental Osteology, Department of Maxillofacial Surgery, University Medical Center, University of Goettingen, Goettingen, Germany; 2 Department of Neuro- and Sensory Physiology, University Medical Center, University of Goettingen, Goettingen, Germany; Centro Cardiologico Monzino, Italy

## Abstract

**Background:**

The octamer-binding transcription factor 4 (Oct4) was originally described as a marker of embryonic stem cells. Recently, the role of Oct4 as a key regulator in pluripotency was shown by its ability to reprogram somatic cells *in vitro*, either alone or in concert with other factors. While artificial induction of pluripotency using transcription factors is possible in mammalian cell culture, it remains unknown whether a potential natural transfer mechanism might be of functional relevance *in vivo*. The stem cell based regeneration of deer antlers is a unique model for rapid and complete tissue regeneration in mammals and therefore most suitable to study such mechanisms. Here, the transfer of pluripotency factors from resident stem cell niche cells to differentiated cells could recruit more stem cells and start rapid tissue regeneration.

**Methodology/Principal Findings:**

We report on the ability of STRO-1^+^ deer antlerogenic mesenchymal stem cells (DaMSCs) to transport Oct4 via direct cell-to-cell connections. Upon cultivation in stem cell expansion medium, we observed nuclear Oct4 expression in nearly all cells. A number of these cells exhibit Oct4 expression not only in the nucleus, but also with perinuclear localisation and within far-ranging intercellular connections. Furthermore, many cells showed intercellular connections containing both F-actin and α-tubulin and through which transport could be observed. To proof that intercellular Oct4-transfer has functional consequences in recipient cells we used a co-culture approach with STRO-1^+^ DaMSCs and a murine embryonic fibroblast indicator cell line (Oct4-GFP MEF). In this cell line a reporter gene (GFP) under the control of an Oct4 responsive element is only expressed in the presence of Oct4. GFP expression in Oct4-GFP cells started after 24 hours of co-culture providing evidence of Oct4 transfer from STRO-1^+^ DaMSCs to Oct4-GFP MEF target cells.

**Conclusions:**

Our findings indicate a possible mechanism for the expansion of a resident stem cell niche by induction of pluripotency in surrounding non-niche cells via transfer of transcription factors through intercellular connections. This provides a new approach to explain the rapid annual antler regrowth.

## Introduction

Stem cells have the capacity of self-renewal and generation of differentiated cells to replenish lost or damaged tissue. In adult mammals, stem cell niches play an essential role in regulating these properties [1]–[3]. The availability of induced pluripotent stem cells by genetic transfer of pluripotency factors enabled new approaches in stem cell research and personalized medicine [4]–[8]. Summarizing recent literature, it has been established that mammalian somatic cells can be reprogrammed to pluripotent states by exogenous expression of the transcription factors Oct4, Klf4, Sox2 and c-Myc [7], [9]–[12]. In addition, it has been demonstrated that induced pluripotent stem cells could also be generated with fewer exogenous transcription factors supported by endogenous expression of reprogramming factors and/or synthetic small molecules [13]–[17]. The octamer-binding transcription factor 4 (Oct4, also known as POU5F1), originally described as a marker of embryonic stem cells [18], plays a key role in the induction of pluripotency as shown by its ability to reprogram somatic cells, either alone [16], [19], [20] or in concert with other factors [9], [13], [17], [21], [22]. Moreover, the expression of transcription factor Oct4 is thought to be important even in adult human stem cells [23]–[26]. It is also discussed whether Oct4 is a master regulatory gene in cell pluripotency and may serve as a pluripotency determinant in reprogramming [14]. Thus, from the current point of view Oct4 is an essential reprogramming factor, enabling mature somatic cells to revert to an embryonic stem cell (ES) – like state, but it does not appear to be required for somatic stem cell self-renewal [27]. Ralston and Rossant (2010) described that functional differences in response to Oct4 exist between adult and ES cells, but that the molecular basis for these differences is not understood.

Zhou et al. (2009), among others, demonstrated that somatic cells can be fully reprogrammed into pluripotent cells by direct delivery of reprogramming proteins *in vitro* [*6*]. Given that *in vitro* induction of pluripotency using defined factors is possible in mammalian cell culture, the question remains whether an analogous mechanism might be of functional relevance *in vivo*. The idea of an *in vivo* induction of pluripotency requires a route of intercellular communication between niche and non-niche cells adequate for transmission of transcription factors like Oct4. Recently, the discovery of membrane nanotubes provided the basis for a cell-to-cell transport of intracellular material [28]–[30]. Zani and Edelman (2010) reviewed the literature about cellular bridges as putative routes for intercellular communication and cell migration and concluded that such bridges provide the potential for directly affecting a greater area of the surrounding biological environment [31]. Regarding antler regeneration, this would be a prerequisite for the expansion of a stem cell niche within solid tissue. Niu et al. (2009) reported on the transfer of cytoplasmic proteins between multiple cell types via transient membrane fusion. They suggest that this phenomenon plays an important role in interactions between stem cells and adjacent somatic cells [32].

In contrast to some lower vertebrates mammals do not possess the capability for appendage regeneration [33]. In this context, we previously described that in deer antler not only limited tissue regeneration but also replacement of a complete appendage in a postnatal mammal can occur as a stem cell-based process [34]. Understanding the mechanisms this unique model for rapid tissue formation [33], [35] may provide utilities to promote tissue regeneration in humans. Our previous results support the view that a stem cell niche mainly consisting of STRO-1^+^ cells and located in the pedicle periosteum provides the basis for the annual antler regeneration [34]. These STRO-1^+^ cells possess the capability to differentiate into cells of the osteogenic, the adipogenic, or the chondrogenic lineage. In the case of the annual regrowth of deer antlers, expansion of the stem cell niche by induction of pluripotency in surrounding non-niche cells might be the key to understand by which means a small number of resident stem cells is able to accomplish such a rapid tissue formation (*up to 1–2 cm per day* [35]).

Since expression of Oct4 in human marrow stromal cells (hMSCs) was described previously [23], [36] we analysed STRO-1^+^ cells derived from the pedicle periosteum of fallow deer (*Dama dama*) and red deer (*Cervus elaphus*) for Oct4 expression.

## Results and Discussion

### STRO-1^+^ DaMSCs express the transcription factor Oct4

**Figure 1:**
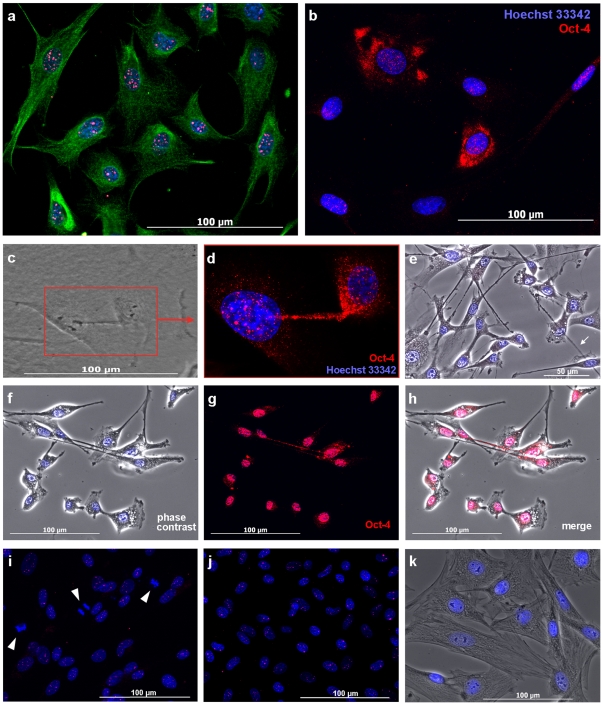
Intracellular distribution and intercellular transport of Oct4 in STRO-1^+^ DaMSC cultures. (**a**) DaMSCs 24 hours after cell sorting and subsequent cultivation in stem cell expansion medium. Antibodies against STRO-1 (pseudo-coloured green) and Oct4 (pseudo-coloured red) were used. Nuclei were counter-stained with Hoechst 33342 (pseudo-coloured blue). Cultured cells showing the surface marker STRO-1 and the transcription factor Oct4 is largely confined to the nuclei. (**b**) Single-staining with Oct4. Elevated Oct4 expression can be typically observed around 2–4 days after sorting and then about 10% of the cells exhibit intensive Oct4 staining with perinuclear localisation. (**c**) Phase-contrast picture of two STRO-1^+^ cells showing a distinct cell-to-cell connection leading from one cell directly to the adjacent cell. The detail (**d**) demonstrates the same cells stained with Oct4 antibody (pseudo-coloured red). Oct4 immunostaining is visible perinuclear and within the cell-to-cell connection. One cell exhibits a higher Oct4 concentration which is enriched towards the membranous tube. (**e**) Phase contrast picture demonstrating that in the majority of cases the intercellular connections are not attached to the substrate. The white arrow point to a floating long-distance cell connection. (**f**) Phase-contrast picture of STRO-1^+^ cells linked by thin cell connections (nuclei counterstained with Hoechst 33342). (**g**) Oct4 immunostaining (pseudo-coloured red) indicates the presence of Oct4 protein in the nuclei of the cells as well as inside the cell-to-cell connections. (**h**) Merged image of pictures (f) and (g). (**i**) In mitotic stages (arrow heads) Oct4 staining is absent. (**j**) About 6–8 days after sorting Oct4 staining is usually limited again to the nuclei. (**k**) Negative control. Staining without primary antibodies.

**Figure 2:**
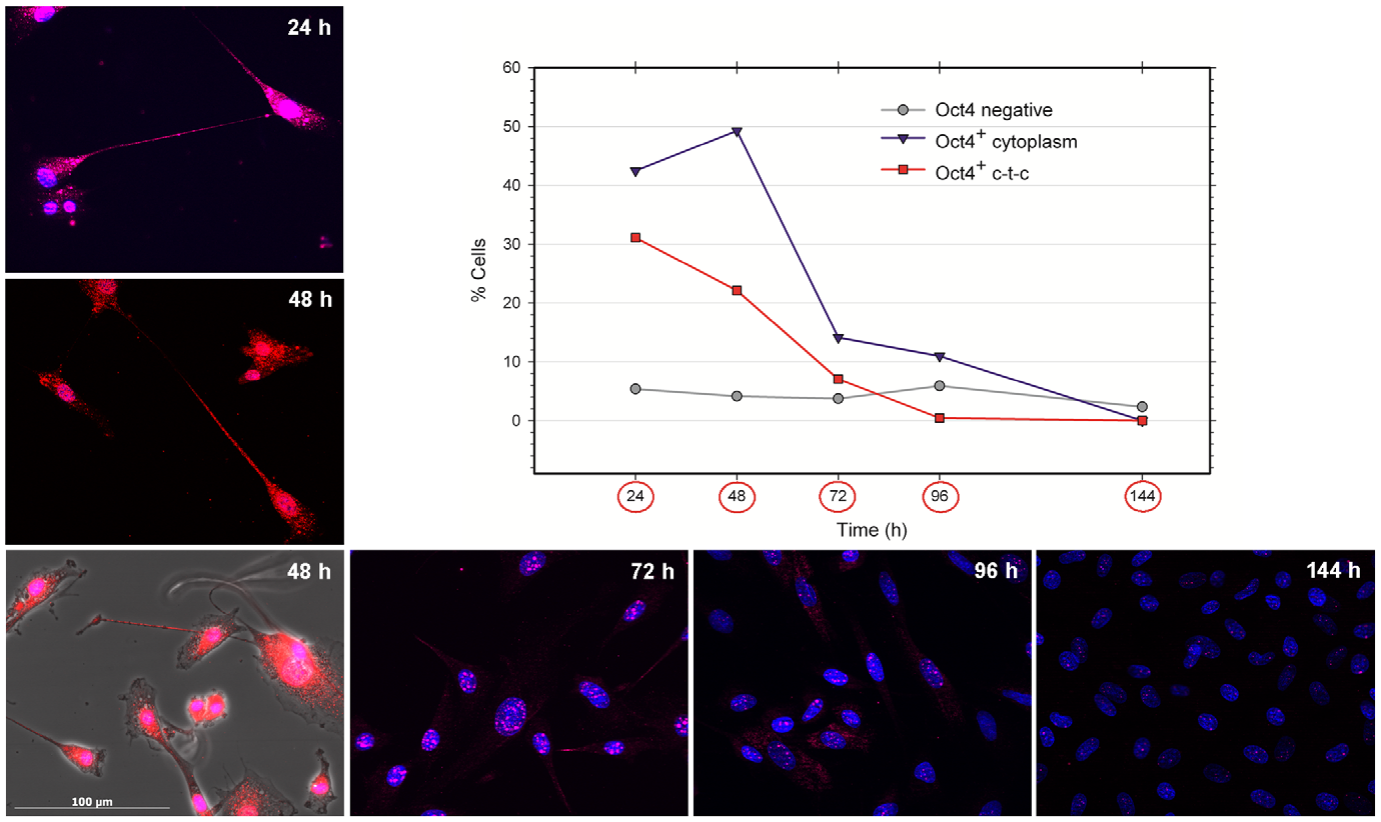
Distribution of Oct4 within immunostained STRO-1^+^ DaMSC cultures. A total of 165 microscopic images from immunostained STRO-1^+^ DaMSCs were analysed. The pictures were taken with high optical magnification during 0–144 hours of cultivation and are based on about 35 different STRO-1^+^ DaMSC cultures. A total of 1688 STRO-1^+^ DaMSCs were evaluated by counting Oct4 positive and negative cells as well as by counting cells containing Oct4 inside the cytoplasm and cells with Oct4 positive c-t-c connections. The amount of cells meeting the different criteria is expressed as percentage of all 1688 cells (diagram). The pictures demonstrate selected examples for each evaluated point of time. Except for one picture (48 h, left side) all pictures are without phase-contrast to enable better visibility of Oct4 expression. Antibodies against Oct4 (pseudo-coloured red) were used. Nuclei were counter-stained with Hoechst 33342 (pseudo-coloured blue).

The staining pattern for Oct4 in STRO-1^+^ DaMSCs as distinct dots within the nucleus (Fig. 1a) was consistent with the expected expression pattern of a transcription factor. The amount of positive Oct4 staining was variable, ranging from only a few dots to a more comprehensive staining of the nucleus (Fig. 1a, g). Notably, among these cells we observed a number of cells that showed distinct Oct4 staining outside the nucleus (Fig. 1b). The Oct4 expression in STRO-1^+^ DaMSCs exhibited a time-dependent regulation. Our experiments revealed elevated Oct4 expression perinuclear and within cell-to-cell (c-t-c) connections about 2–4 days after sorting (Fig. 1b, d, g; 2). Later on, cytoplasmic Oct4 staining is absent and Oct4 distribution resembles the situation which had been observed 1–2 days after sorting (Fig. 1j; 2). Statistical analyses of 165 microscopic images of Oct4 immunostained STRO-1^+^ DaMSCs based on more than 35 different cultures supported these findings (Fig. 2). 24–48 hours after cell sorting between 42–49% of the seeded STRO-1^+^ DaMSCs exhibited nuclear and cytoplasmic Oct4 expression. After 96 hours of cultivation the cytoplasmic expression decreased to 11%. The amount of cells showing Oct4 expression within c-t-c connections decreased from 31% (24 hours) to 0.5% after 96 hours of cultivation. Oct4 expression within cytoplasm and c-t-c connections was no longer detectable after 144 hours of cultivation. In addition, we observed that the amount of Oct4 expression inside the nuclei of STRO-1^+^ DaMSCs decreased also with time in culture (Fig. 2). Oct4 staining could not be detected in cells during mitotic stages (Fig. 1i). Since we detected Oct4 expression only in STRO-1^+^ sorted cells, we assume that sorting mimics a signal to activate Oct4 expression. This signal could either be the binding of the STRO-1 antibody or the loss of an inhibiting factor previously provided by cells of the mixed culture. This assumption needs to be clarified in further experiments.

**Figure 3:**
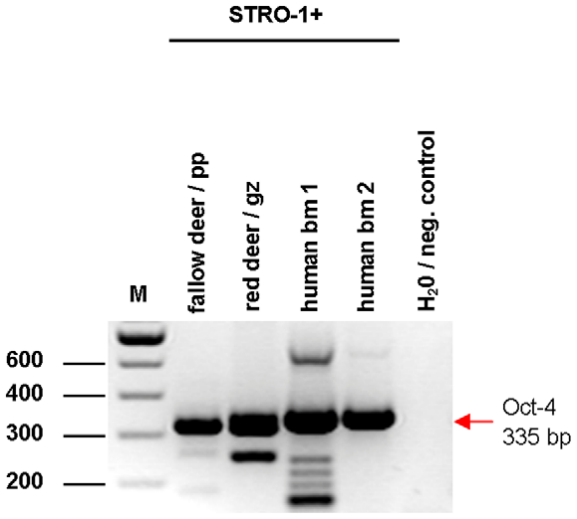
RT-PCR analyses of Oct4 expression in STRO-1^+^ cells. STRO-1^+^ cells derived from cultured pedicle periost (pp) of regenerating fallow deer antlers, growth zone (gz) of regenerating red deer antlers and human bone marrow (bm) from iliac crest biopsies were used as source for cDNA. PCR on these samples yielded bands representing the 3′-end of exon1 (335 bp). The bands were extracted and sequenced, demonstrating a very high homology of Oct4 of deer and human origin.

Recently, concerns were raised over the correctness and relevancy of findings regarding Oct4 expression in cells other than embryonic stem cells because many Oct4 data were generated without distinguishing between Oct4 transcript variants [37]. Apart from that, the existence of pseudogenes contributes to the complexity in the human system. Some pseudogenes are transcribed and at least one (POU5F1P1, NM_001159542) is even translated into protein and able to act as a transcriptional activator similar to but weaker as Oct4A [38]. We used RT-PCR and sequence analysis to verify the Oct4 identity in STRO-1^+^ DaMSCs of adult fallow and red deer stags. Since the Oct4 sequence in deer is not known, we rely on corresponding sequences derived from related species like cattle. Therefore, we chose primers binding to sections conserved between the bovine and the human Oct4 sequences. We included human cDNA from bone marrow aspirate cultures to confirm that the primers were also able to detect Oct4 expression in adult human mesenchymal stem/progenitor cells [24], [36]. In all samples a PCR fragment of the expected size was detected (Fig. 3). As two transcript variants of Oct4 are known in humans, our primers were specific to the first exon which is present only in Oct4A (NM_002701). Thereby, we were able to exclude the second transcript variant Oct4B (NM_203289). This variant is considered to be irrelevant for pluripotency in the human system [39] and is suspected to account for some of the examples of Oct4 expression in adult stem cells reported in literature [37]. Therefore, we further examined the PCR fragments obtained from all investigated samples by sequence analyses. The human PCR fragments contained certain point mutations indicative for the Oct4 pseudogenes POU5F1P1 and POU5F1P3 (NG_005793). The investigated PCR fragments from deer samples exhibited a high homology (up to 98%) with the known Oct4 sequence in cattle (Bos taurus, NM_174580), the phylogenetically nearest relative represented in the NCBI database, and a homology of up to 78% with human Oct4A. Information about possible pseudogenes in deer is not available.

### Cell-to-cell connections between STRO-1^+^ DaMSCs

**Figure 4:**
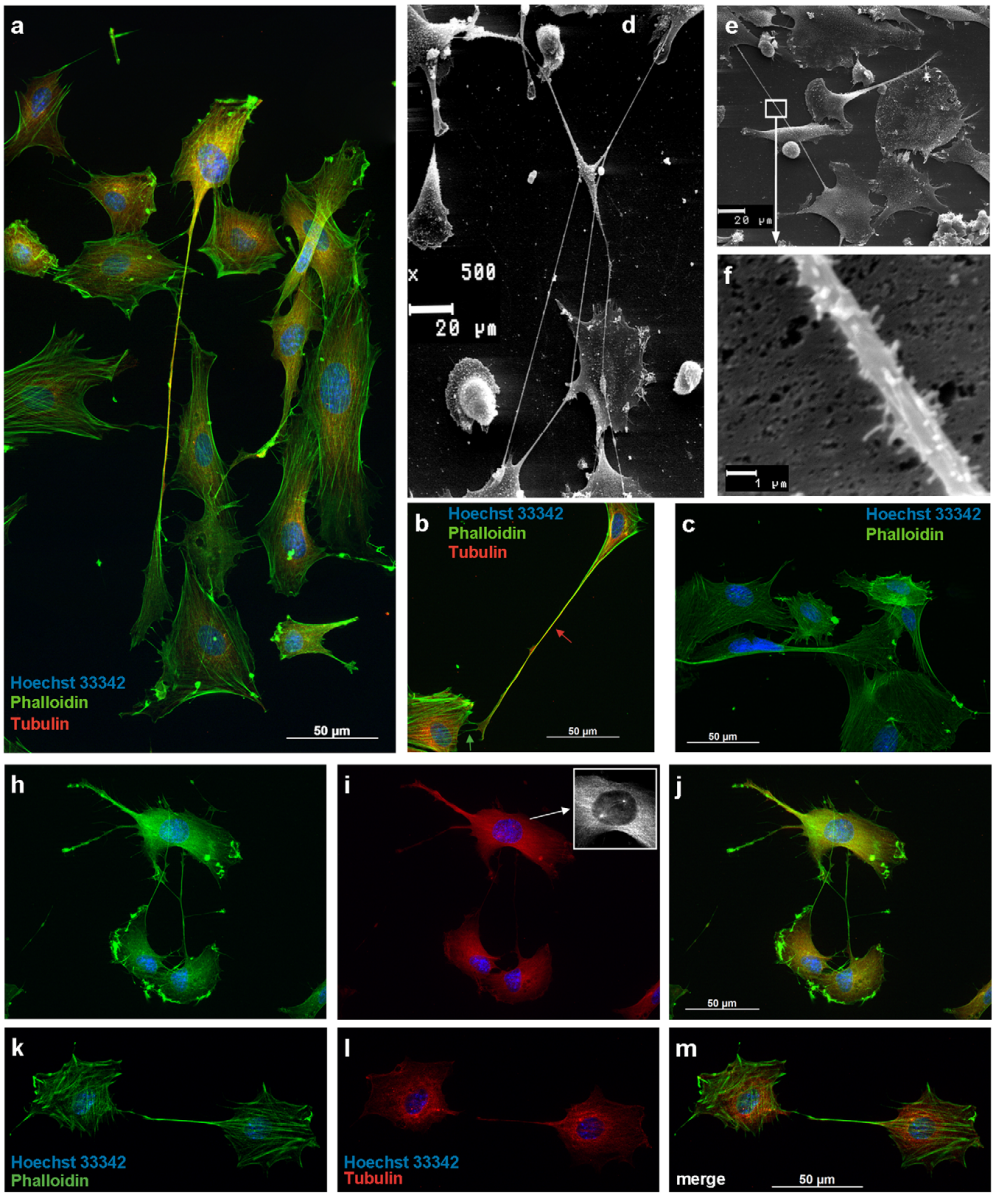
Cytoskeletal elements of STRO-1^+^ DaMSCs and long-distance cell-to-cell connections (TNTs). (**a,b**) Different cell-to-cell connections between STRO-1^+^ cells. Intercellular connections are able to bridge long distances even across neighbouring cells (Phalloidin/Tubulin staining, merged images). (**b**) Thick tubes (diameter >0.4 µm, example is marked by red arrow) contain F-actin and α-tubulin, whereas thin tubes (diameter <0.4 µm, example is marked by green arrow) contain only F-actin. (**c**) Negative control, staining without Tubulin antibody. (**d–f**) Scanning electron microscope pictures (SEM) of a mixed culture of antlerogenic cells. Cells form long connections across neighbouring cells (**d,e**) and very high magnification (**f**) proves that the surface of TNTs exhibit small appendages; long-distance connections are also possible between morphologically distinguishable cell types (**d**). (**h–m**) Multichannel pictures of Phalloidin (**h,k**) and Tubulin (**i,l**) stained DaMSCs demonstrate that their intercellular connections continuously consist of F-actin but only partially of microtubules. The visible spindle apparatus (**i**, detail enlargement) point to an initiating cell division and provides evidence that the used α-tubulin antibody is also efficient in DaMSCs. (**j,m**). Merged Images.

While Oct4 in DaMSCs was predominantly found in the nucleus, some cells showed enriched cytoplasmic staining in the perinuclear region as well as within long membrane extensions (Fig. 1 b, d, g and 2). Increasing cytoplasmic Oct4 synthesis is a prerequisite to enable transfer of Oct4 from donor cells to recipients, Oct4 genes must be activated in the donor cells and then a simultaneously Oct4 expression must be also detectable within the nuclei. We never found Oct4 staining in cytoplasmic areas alone and therefore our results thoroughly point to a functional relevance of the Oct4 accumulation within cytoplasm and intercellular connections. Long-distance c-t-c connections have been observed regularly in our antler cell cultures and span distances of 50 to >300 µm (Fig. 4). In many cases these intercellular connections have no contact to the substrate (Fig. 1e, f; 4d–f). Staining against F-actin and α-tubulin (Fig. 4a–c and h–m) suggests that these membrane extensions are comparable to a new kind of c-t-c communication, referred to as tunnelling nanotubes (TNTs) which were first described by Rustom et al. [40]. TNTs were recently identified in a growing number of cell types like progenitor cells, immune cells, tumour cells or epithelial cells [28]–[30], [41]. Oenfelt et al. (2006) investigated membrane nanotubes between human macrophages and distinguished between two classes of nanotubular cell connections by their cytoskeletal structure and their functional properties [42]. They described that thin membrane nanotubes contain only F-actin, whereas thicker ones (diameter: >0.7 µm) contain both F-actin and microtubules. Consistent with the literature [29], [40], [42], [43] we also observed different types of nanotubular cell connections: (1) Thin membrane tubes with a diameter of 0.31±0.1 µm (mean ± SD, n = 28) containing only F-actin and (2) thicker membrane tubes with a diameter >0.68±0.2 µm (mean ± SD, n = 43) containing F-actin and α-tubulin (Fig. 3b, h–m). We noticed that sometimes the same c-t-c connection may contain both F-actin and α-tubulin only within thicker parts of the membrane tube whereas tubulin could not be detected inside thinner regions. Therefore, we assume that similar to previously described observations [29] the primary formation of interconnections between DaMSCs (Fig. 4h–m) seems also to be most probably driven by actin polymerization.

**Figure 5:**
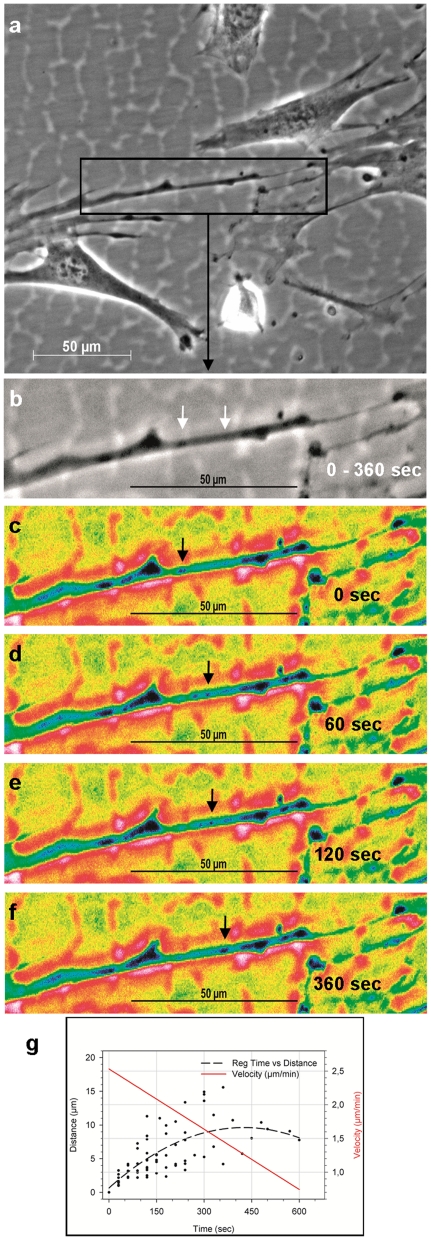
Cargo transport via intercellular bridges. (**a**) Phase-contrast picture of STRO-1^+^ DaMSCs showing cell-to-cell connections mediated by membrane extensions and nanotubular structures (TNTs). (**b**) Detail of figure (a). Membranous tube containing cargo. The distance covered by cargo within 360 seconds is marked by arrows. (**c–f**) Pseudo-coloured pictures demonstrating cargo transport within 360 seconds. To exemplify cargo transport one specific transported cellular component is marked by arrows. (**g**) The graph represents the velocity of different transported particles (n = 68). The measured values of the particle's running distance (l) are plotted against the time (t) elapsed. The dashed line represents the regression (reg) of these values (l = −0.497e^5^t^2^+0.04t+0.66; n = 68; r = 0.68). The first derivative of the regression (Δl/Δt) shows the particle's velocity (µm/min).

TNT-like structures are known to transport cargo of various sizes ranging from proteins [44], over viral particles [45] to entire organelles [46] between cells. Gerdes and Carvalho (2008) reckon that the growing number of reports on TNT-like structures point to an underlying general principle of c-t-c communication [29]. Fast motion video recordings of STRO-1^+^ DaMSCs revealed unidirectional cargo movement within c-t-c connected membrane tubes (Fig. 5a–f, Supporting Information Video S1 and Video S2). The observed particles moved at a rate of 0.7–2.5 µm/min and decelerated gradually with time and distance (Fig. 5g). A comparable velocity was reported for an actin-based, unidirectional vesicle transfer in neuronal cells via TNTs [40]. As we observed dot-like Oct4 staining within cell connections between STRO-1^+^ DaMSCs (Fig. 1d, g; 2) we tried to detect transport of Oct4 within these TNT-like structures.

**Figure 6:**
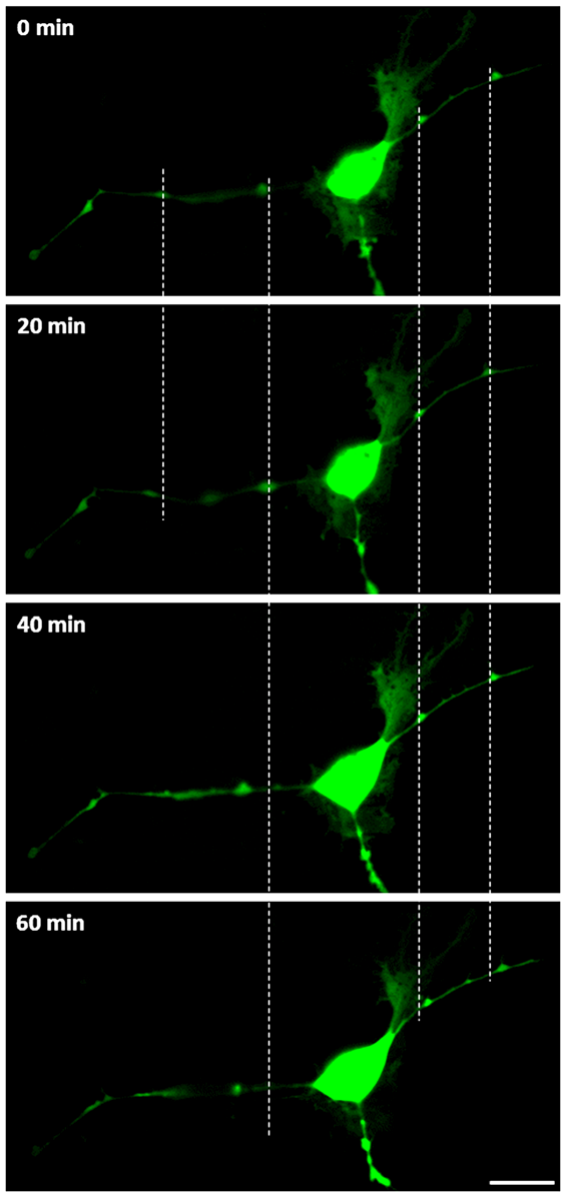
Transport of Oct4 fusion protein via cytoplasmic connections. (**a–d**) Time series pictures of a transfected STRO-1^+^ DaMSC. The GFP fluorescence of the Oct4 fusion protein is visible within the cytoplasm as well as inside the membrane tubes. Membrane dilatations (gondolas) filled with Oct4 fusion proteins are moving away from the cellular body. Starting points are marked with white dashed lines. Scale bar = 20 µm.

Since antibody staining of intracellular epitopes is not possible in living cells, first of all we used a transfection approach with a plasmid encoding a fluorescence labelled Oct4 (pLM-vexGFP-Oct4 as Plasmid 22240 from Addgene). In transfected STRO-1^+^ DaMSCs we observed transport of the Oct4 fusion protein via cytoplasmic connections (Fig. 6) and the fusion protein was also frequently visible in dilatations of the membrane tubes. Such dilatations were reported previously and named gondolas [47]. The Oct4 loaded gondolas moved unidirectional (away from the cellular body) along the c-t-c connections and the transport appears similar to mechanisms of intercellular communication described e.g. for urothelial cell lines [47]. This indicates that a mechanism enabling transfer of transcription factors exists in mammalian cells.

### Transfer of Oct4 from STRO-1^+^ DaMSCs to Oct4/GFP-MEF indicator cells

**Figure 7:**
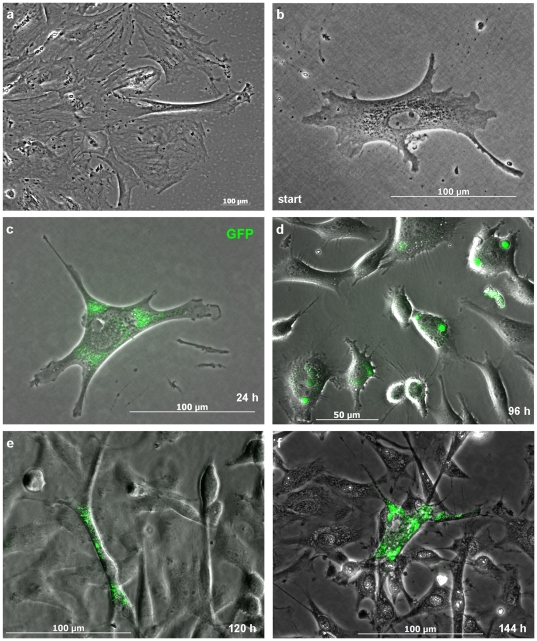
Mixed culture of Oct4-GFP MEF cells and STRO-1^+^ DaMSCs. (**a**) Colony of Oct4-GFP MEF cells without any GFP expression. (**b**) Individual Oct4-GFP MEF cell after 24 hours of pre-cultivation in stem cell expansion medium on the eve of co-cultivation. (**c**) The same Oct4-GFP MEF cell after 24 hours of co-cultivation with STRO-1^+^ DaMSCs (interacting DaMSCs are outside of the display window). GFP expression (pseudo-coloured green) is visible within the cytoplasm. (**d**) Mixed culture after 96 hours of co-cultivation. More GFP^+^ cells interacting with GFP^−^ cells are visible. (**e**) Cell with distinct GFP expression after 120 hours of co-cultivation. At that time GFP^+^ cells interact continuously with GFP^−^ cells and are well integrated into the forming multilayer. (**f**) After 144 hours of co-cultivation some cells exhibit widely distributed intracellular GFP expression. (a,b,c,f = phase contrast pictures; d,e = varel contrast pictures; a–e = pictures of living cultures, f = fixed cells).

After showing that transcription factor Oct4 is transported between DaMSCs along TNT-like intercellular connections, we wondered whether such a transport has functional consequences, e.g. whether the transcription factor can enter other cells and can induce transcription in recipient cells. For further analyses we used the murine Oct4 reprogramming control cell line Stemgent™ Oct4-GFP MEF [48], in which GFP expression is under the control of an endogenous Oct4 promoter. Using murine and deer cells should not cause problems considering the recent literature. Ho et al. (2011) realised that the diversity of cell types and species that have been reprogrammed and the general applicability of the four original reprogramming factors (Oct4, Sox2, c-Myc and Klf4) suggests a generic fashion in which the four factors act [49]. The authors concluded that there probably is no cell type-specific barrier that cannot be overcome by the action of the reprogramming factors leading to an evolutionary conserved pluripotency network. Interestingly, they mentioned that the starting cell type can alter the dependence on the reprogramming factors, efficiency, and kinetics *in vitro*; and the authors hypothesised that all somatic cells may be amendable to reprogramming, but more undifferentiated cells in the population have a higher probability to overcome reprogramming barriers [49]. Their theory is supported by another study about the reprogramming of human somatic cells which used various neonatal somatic cells. This study revealed differences in reprogrammability between the investigated cell types [50] and the results also point to different kinetics of reprogramming suggesting that some cell types might be more amenable to reprogramming than others. We co-cultivated Oct4-GFP MEF cells together with STRO-1^+^ DaMSCs and monitored for GFP expression. Since only about 10% of STRO-1^+^ DaMSCs exhibit distinct Oct4 staining outside the nucleus and within c-t-c connections we carried out the co-culture experiments with a DaMSC surplus to increase the chance of an Oct4 transfer. Neither indicator cells (Fig. 7a, b), nor STRO-1^+^ DaMSCs exhibited GFP expression when cultured alone. We detected GFP expression in a few MEF cells already 24 hours after co-cultivation with STRO-1^+^ DaMSCs (Fig. 7c). Increasing intracellular GFP expression as well as increasing numbers of GFP^+^ cells could be observed within the following days (Fig. 7d–f). Although our co-cultures consisted of cell-types derived from two different animal species, increasing c-t-c interactions could be observed (Fig. 7d) and GFP^+^ cells became well integrated into the forming multilayer (Fig. 7e, f).

Due to the fact that our co-culture experiments resulted in considerable GFP expression in Oct4-GFP MEF cells already after 24 hours of co-cultivation, there is evidence to suggest that they received the transcription factor Oct4 from the STRO-1^+^ DaMSCs. Our results demonstrate Oct4 transfer and onset of GFP expression in Oct4-GFP MEF recipient cells on a single-cell level. The observation of single cells provides an opportunity to detect earliest reactions of recipient cells.

Considering recent studies, it seems unsurprising that the target cells in our experiments react immediately to the transferred Oct4. Experiments from Li et al. (2011) with MEF cells suggested that after initiation reprogramming is independent of continuous exogenous Oct4 expression [14]. Based on these findings it seems conceivable that very small amounts of Oct4 protein transported via TNTs are sufficient to initiate an immediate reaction in the recipient cell.

Although our experiments left no doubt that STRO-1^+^ DaMSCs released Oct4 directly via c-t-c connections, we also tried to exclude further possible alternatives, for instance a conceivable intercellular transfer of Oct4 via exosomes [51]. For these purposes we carried out experiments using supernatants of our STRO-1^+^ DaMSC cultures. The used preparation method ensured that the collected supernatants should contain exosomes or related types of vesicular carriers of membrane and cytosolic components potentially secreted by STRO-1^+^ DaMSCs. In contrast to our co-cultures, Oct4-GFP MEF recipient cells cultivated up to 120 hours with supernatants derived from STRO-1^+^ DaMSC cultures showed no GFP expression, neither with supernatants collected between 0–48 hours of cultivation nor with supernatants collected between 48–120 hours of cultivation. Therefore, we conclude that in STRO-1^+^ DaMSCs a potential c-t-c transfer of Oct4 via exosomes seems to be unlikely.

Regarding our co-culture experiments, the onset of GFP expression from the endogenous Oct4 promoter represents an activation of this important pluripotency marker gene and may point to an initiation of reprogramming in the Oct4-GFP MEF indicator cell line. However, we would like to point out that our experiments were only designed to prove a possible transfer of the transcription factor Oct4 from STRO-1^+^ DaMSCs to potential recipient cells. Two questions remain, (1) whether Oct4 acts alone or in concert with different co-factors and (2) whether the Oct4-dependent initiation of transcription in Oct4-GFP MEF cells in our co-cultures induces real reprogramming or mere proliferation.

With regard to deer antler regeneration, we suppose that a special situation exists within the deer's pedicle periosteum. In our hypothesis, a persistent stem cell niche mainly consisting of STRO-1^+^ DaMSCs might be surrounded by more or less differentiated somatic cells amenable to the transcription factor network that regulates pluripotency. Once a year, this niche becomes powered by as yet unknown factors and the DaMSCs begin with self-renewal and simultaneously, a contingent of these cells starts to transfer Oct4 and/or other factors/co-factors to the surrounding environment to enable immediate and rapid tissue regeneration. In principle, the expression of Oct4 in STRO-1^+^ deer antler stem cells suggests that they possess even more primordial characteristics and a potential beyond mesenchymal cell types. Furthermore, the functional effect of the Oct4 protein across species barriers observed in our co-cultures supports the theory of an evolutionary conserved pluripotency network. If intercellular transfer of transcription factors is also a basic principle in other adult mammalian stem cells, it might be of fundamental relevancy with regard to the interpretation of stem cell niche capabilities.

### Conclusions

In this study we have found that the oct4 gene and Oct4 protein are expressed in STRO-1^+^ DaMSCs. These cells are involved in annual appendage regeneration in deer [34]. Moreover, Oct4 positive DaMSCs are able to initiate transcription in an Oct4-GFP MEF indicator cell line mediated by intercellular Oct4 transfer via TNT-like cell connections. This substantiates our hypothesis that deer antler stem cells can recruit surrounding cells for rapid tissue regeneration by transfer of transcription factor Oct4. Since the number of stem cells persisting in a niche is limited we believe that this expansion might be a prerequisite to enable such a rapid tissue formation that accomplishes complete appendage regeneration in deer.

## Materials and Methods

### Ethics Statement

All tissue samples were taken in compliance with the institutional guidelines on animal husbandry and care/welfare of the University Medical Center in Goettingen (Central Animal Facility) and the Institute for Wildlife Biology and Game Management (Faculty of Forest Sciences and Forest Ecology), University of Goettingen, Germany. The study was approved by the competent district government (Braunschweig, Germany) – permit numbers: 604.42502/01-21.96 and 604.42502/01-22.96.

Sampling of human bone marrow aspirates was carried out in compliance with the written informed consent of the patients as well as with the approval of the ethics committee of the University Medical Center in Goettingen (permit number: 15/10/01).

### Cell culture

Three adult fallow deer, aged between 4 and 6 years, were sacrificed within four weeks after the onset of antler regrowth. In addition, tissue samples derived from the growth zone of regenerating antlers were taken from four red deer stags about 10–14 days after antler casting. Primary tissue obtained from the pedicle periosteum and the antler growth zone was cultivated in Dulbecco's modified eagle medium (DMEM) to get a mixed culture consisting of adherent cells. Upon reaching confluence STRO-1^+^ progenitor cells were isolated as described previously [34] using the MACS® technology (Miltenyi Biotech Inc., Bergisch Gladbach, Germany) according to the manufacturer's instructions. Subsequently, the STRO-1^+^ cells were cultivated for 24–96 hours in NH-Expansion Medium (Miltenyi Biotec) followed by fixation with 70% ethanol. The Lymphoprep™ (Axis-Shield PoC AS, Norway) technique was used to prepare human bone marrow aspirates. Subsequently, cultivation and isolation of STRO-1^+^ cells was carried out similar to primary tissue obtained from the pedicle periosteum and the antler growth zone. Cell numbers in the cultures were determined by using an electronic cell counter system (CASY®, Schaerfe System, Reutlingen, Germany).

### Immunocytochemistry

Immunostaining was performed with anti-human STRO-1 (MAB 1038, R&D Systems) and anti-human Oct-3/4 (H-34:sc-9081, polyclonal, Santa Cruz Biotechnology Inc.), using FITC Goat anti Mouse IgG/IgM (BD Pharmingen, Germany) and Northern Lights™ Anti-rabbit IgG-NL557 (NL004, R&D Systems) as secondary antibodies, respectively. Primary antibodies were diluted 1∶50 and secondary antibodies 1∶100.

#### Detailed protocol

**1)** Cells were fixed in 70% ethanol. **2)** Cell membranes were permeabilised with 0.5–1 ml iced methanol for 15–30 minutes at −20°C. **3)** Unspecific antibody binding was blocked by using Triton X 100+10% swine serum for 20 minutes at room temperature (RT). **4)** Samples were incubated overnight with diluted primary antibodies at 4°C. **5)** Incubation with fluorescence-conjugated secondary antibodies was carried out in the dark for 90 minutes at RT. **6)** The nuclei were counter-stained with Hoechst 33342 fluorescent dye (Invitrogen, Germany) for 10 minutes (dilution: 1∶1000, incubation in the dark at RT). **7)** The slides were prepared with FluorPreserve™ Reagent (Calbiochem®, Germany). With the exception of work step 3 (blocking step), after each individual operation samples were rinsed at least three times with PBS buffer.

For fluorescence pictures a Zeiss Axiovert 200 M microscope equipped with an ApoTome® (Carl Zeiss, Germany) was used. Preparation for scanning electron microscopy (SEM) was described elsewhere [34]. Sigma Plot® (Erkrath, Germany) was used for statistical analyses.

### Staining of cytoskeletal elements

#### Detailed protocol of α-tubulin staining

**1)** Cells were fixed in 4% paraformaldehyde. **2)** Cell membranes were permeabilised with 0.1% TritonX100 for 15 minutes at RT. **3)** Unspecific antibody binding was blocked by using 1% BSA for 30 minutes. **4)** Samples were incubated overnight with an α-tubulin (B-5-1-2) primary antibody (Santa Cruz, sc-23948) at 4°C (dilution: 1∶100–1∶500). **5)** Incubation with a fluorescence-conjugated secondary antibody [Alexa Fluor® 546 goat anti-mouse IgG (H+L), Invitrogen A11003] was carried out in the dark for 90 minutes at RT (dilution: 1∶1000). **6)** The nuclei were counter-stained with Hoechst 33342 fluorescent dye (Invitrogen, Germany) for 10 minutes (dilution: 1∶1000, incubation in the dark). **7)** The slides were prepared with FluorPreserve™ Reagent (Calbiochem®, Germany). With the exception of work step 3 (blocking step), after each individual operation samples were rinsed at least three times with PBS buffer.

F-actin filament staining was performed using a fluorescein isothiocyanate (FITC) labelled Phalloidin (catalogue number P5282, Sigma-Aldrich Inc., USA) according to the manufacturer's staining protocol.

### RT-PCR

RNA was isolated from frozen cell pellets using the RNeasy® Mini Kit (Qiagen, Hilden, Germany) according to the manufacturer's instructions. After lysis the cells were homogenized with QIAshredder™ columns (Qiagen, Hilden, Germany).To avoid contamination with genomic DNA an on-column digestion with RNase-free DNase (Qiagen, Hilden, Germany) was performed. 60–200 ng of total RNA were reverse transcribed with the iScript™ cDNA Synthesis Kit (Bio-Rad, Hercules, California). PCR was performed using the GoTaq® Green Master Mix (Promega, Mannheim, Germany) and 1 µl of cDNA was used as template in each reaction. Primer sequences specific for the first exon were used (Oct4for: 5′-CTGGGTTGATCCTCGGACCT-3′; Oct4rev [37]: 5′-TGATGTCCTGGGACTCCTC-3′) to detect Oct4 expression and the length of the resulting PCR-fragment was 335 bp. Sequence analyses were performed at Sequence Laboratories (Seqlab), Goettingen.

### Transfection

STRO-1^+^ DaMSCs cells were transfected using Promofectine (Promocell) when 80% confluent. 1 µg of the pLM-vexGFP-Oct4 plasmid was used per well in a 6-well-plate. The transfection was performed overnight according to the manufacturer's instructions. 48 h after transfection the culture medium was exchanged for OptiMem (Invitrogen) in order to minimize the background fluorescence in the microscopic analysis. GFP fluorescence of the Oct4 fusion protein was assessed using a Zeiss LSM 510 Meta microscope.

### Co-culture experiments with MEF cells

Experiments with mixed cultures were carried out using the Stemgent™ Oct4-GFP MEF (P2) reprogramming control cell line (Miltenyi Biotec Inc., Bergisch Gladbach, Germany). Stemgent® Oct4-GFP mouse embryonic fibroblasts (MEF) harbour a GFP reporter gene downstream of exon 5 of the endogenous Oct4 locus [48]. Upregulation of the endogenous Oct4 pluripotency locus results in expression of GFP. Oct4-GFP MEF cells were cultured in MEF culture medium described by the manufacturer's product specification sheet (Stemgent™, San Diego, USA) and observed for 6 weeks to make sure that not any GFP expression is visible in the MEF culture. Subsequently, the GFP-negative MEF cells were trypsinised and seeded into 4-well chambers (Lumox®, 4-well. Ref.No. 94.6150.401, Sarstedt, Germany). The cells were pre-cultivated overnight and STRO-1^+^ DaMSCs were added at the ratio of 7∶1 (STRO-1^+^ DaMSCs/Oct4-GFP MEF cells). All co-culture experiments were carried out in NH-Expansion Medium (Miltenyi Biotec). GFP expression was observed under the fluorescence microscope during 144 hours of cultivation.

### Preparation of supernatants derived from STRO-1^+^ DaMSC cultures

Culture supernatants of STRO-1^+^ DaMSC cultures were collected between 0–48 hours and 48–120 hours of cultivation. The pooled samples were centrifuged at 300× g for 10 minutes and then at 1200× g for 10 minutes to eliminate cells and debris. These values were based on parts of protocols that were used for exosome preparations published by different authors [52], [53]. The centrifuged supernatants were mixed at the rate of 1∶1 with fresh NH-Expansion Medium to be used in Oct4-GFP MEF cultures.

### Experiments with STRO-1^+^ DaMSC supernatants

Different passages (P2 and P3) of Oct4-GFP MEF cells were grown in 2-well culture chambers. After 24 hours of initial cultivation in NH-Expansion Medium cultures were rinsed with PBS and afterwards they received the prepared supernatant/NH-Medium mixtures. Cultivation experiments with STRO-1^+^ DaMSC supernatant samples were carried out up to 120 hours to detect possible GFP-expression in Oct4-GFP MEF cells.

### Measurements and statistical analyses

A time lapse series of images with a time interval of 30 seconds was used to calculate the velocity of the vesicles moving inside the nanotubes. The time (t) elapsed was plotted against the measured values of the vesicle's running distance (l). The regression of the plot represents the distance-time curve and the slope of this curve is the vesicle's velocity. Therefore, we calculated the 1^st^ derivative of the distance-time regression which is the curve of velocity.

To evaluate the distribution of Oct4 within immunostained STRO-1^+^ DaMSCs during 0–144 hours of cultivation we analysed 165 microscopic images taken with high optical magnification. The pictures were based on about 35 different STRO-1^+^ DaMSC cultures. A total of 1688 STRO-1^+^ DaMSCs were evaluated by counting Oct4 positive and negative cells as well as by counting cells containing Oct4 inside the cytoplasm and cells with Oct4 positive c-t-c connections. The amount of cells meeting the different criteria was expressed as percentage of all 1688 cells (Fig. 2).

## Supporting Information

Video S1 Cell-to-cell connection between STRO-1^+^ DaMSCs (equivalent to Figs. 5b–f; detail of Fig. 5a). This fast motion movie (original speed = one picture every 10 seconds) demonstrates unidirectional cargo transport through an intercellular bridge within 360 seconds.
(MP4)

Video S2 Pseudo-coloured version of Video 1 to visualize the transport sequence.
(MP4)

## References

[1] Li L, Xie T (2005). Stem cell niche: structure and function.. AnnuRevCell DevBiol.

[2] Jones DL, Wagers AJ (2008). No place like home: anatomy and function of the stem cell niche.. NatRevMolCell Biol.

[3] Moore KA, Lemischka IR (2006). Stem cells and their niches.. Science.

[4] Vierbuchen T, Ostermeier A, Pang ZP, Kokubu Y, Sudhof TC (2010). Direct conversion of fibroblasts to functional neurons by defined factors.. Nature.

[5] Yamanaka S (2007). Strategies and new developments in the generation of patient-specific pluripotent stem cells.. Cell Stem Cell.

[6] Zhou H, Wu S, Joo JY, Zhu S, Han DW (2009). Generation of induced pluripotent stem cells using recombinant proteins.. Cell Stem Cell.

[7] Takahashi K, Yamanaka S (2006). Induction of pluripotent stem cells from mouse embryonic and adult fibroblast cultures by defined factors.. Cell.

[8] Li C, Zhou J, Shi G, Ma Y, Yang Y (2009). Pluripotency can be rapidly and efficiently induced in human amniotic fluid-derived cells.. Hum Mol Genet.

[9] Park IH, Zhao R, West JA, Yabuuchi A, Huo H (2008). Reprogramming of human somatic cells to pluripotency with defined factors.. Nature.

[10] Takahashi K, Tanabe K, Ohnuki M, Narita M, Ichisaka T (2007). Induction of pluripotent stem cells from adult human fibroblasts by defined factors.. Cell.

[11] Welstead GG, Brambrink T, Jaenisch R (2008). Generating iPS cells from MEFS through forced expression of Sox-2, Oct-4, c-Myc, and Klf4.. J Vis Exp.

[12] Meissner A, Wernig M, Jaenisch R (2007). Direct reprogramming of genetically unmodified fibroblasts into pluripotent stem cells.. Nat Biotechnol.

[13] Kim JB, Zaehres H, Wu G, Gentile L, Ko K (2008). Pluripotent stem cells induced from adult neural stem cells by reprogramming with two factors.. Nature.

[14] Li Y, Zhang Q, Yin X, Yang W, Du Y (2011). Generation of iPSCs from mouse fibroblasts with a single gene, Oct4, and small molecules.. Cell Res.

[15] Yuan X, Wan H, Zhao X, Zhu S, Zhou Q (2011). Brief report: combined chemical treatment enables Oct4-induced reprogramming from mouse embryonic fibroblasts.. Stem Cells.

[16] Kim JB, Sebastiano V, Wu G, Arauzo-Bravo MJ, Sasse P (2009). Oct4-induced pluripotency in adult neural stem cells.. Cell.

[17] Tsai SY, Clavel C, Kim S, Ang YS, Grisanti L (2010). Oct4 and klf4 reprogram dermal papilla cells into induced pluripotent stem cells.. Stem Cells.

[18] Scholer HR, Hatzopoulos AK, Balling R, Suzuki N, Gruss P (1989). A family of octamer-specific proteins present during mouse embryogenesis: evidence for germline-specific expression of an Oct factor.. EMBO J.

[19] Takahashi K, Okita K, Nakagawa M, Yamanaka S (2007). Induction of pluripotent stem cells from fibroblast cultures.. NatProtoc.

[20] Kim JB, Greber B, Arauzo-Bravo MJ, Meyer J, Park KI (2009). Direct reprogramming of human neural stem cells by OCT4.. Nature.

[21] Kaji K, Norrby K, Paca A, Mileikovsky M, Mohseni P (2009). Virus-free induction of pluripotency and subsequent excision of reprogramming factors.. Nature.

[22] Pan C, Lu B, Chen H, Bishop CE (2010). Reprogramming human fibroblasts using HIV-1 TAT recombinant proteins OCT4, SOX2, KLF4 and c-MYC.. MolBiolRep.

[23] Pochampally RR, Smith JR, Ylostalo J, Prockop DJ (2004). Serum deprivation of human marrow stromal cells (hMSCs) selects for a subpopulation of early progenitor cells with enhanced expression of OCT-4 and other embryonic genes.. Blood.

[24] Tai MH, Chang CC, Kiupel M, Webster JD, Olson LK (2005). Oct4 expression in adult human stem cells: evidence in support of the stem cell theory of carcinogenesis.. Carcinogenesis.

[25] Tsai MS, Lee JL, Chang YJ, Hwang SM (2004). Isolation of human multipotent mesenchymal stem cells from second-trimester amniotic fluid using a novel two-stage culture protocol.. HumReprod.

[26] Ji KH, Xiong J, Hu KM, Fan LX, Liu HQ (2008). Simultaneous expression of Oct4 and genes of three germ layers in single cell-derived multipotent adult progenitor cells.. AnnHematol.

[27] Ralston A, Rossant J (2010). The genetics of induced pluripotency.. Reproduction.

[28] Gurke S, Barroso JF, Gerdes HH (2008). The art of cellular communication: tunneling nanotubes bridge the divide.. HistochemCell Biol.

[29] Gerdes HH, Carvalho RN (2008). Intercellular transfer mediated by tunneling nanotubes.. CurrOpinCell Biol.

[30] Davis DM, Sowinski S (2008). Membrane nanotubes: dynamic long-distance connections between animal cells.. NatRevMolCell Biol.

[31] Zani BG, Edelman ER (2010). Cellular bridges: Routes for intercellular communication and cell migration.. CommunIntegrBiol.

[32] Niu X, Gupta K, Yang JT, Shamblott MJ, Levchenko A (2009). Physical transfer of membrane and cytoplasmic components as a general mechanism of cell-cell communication.. JCell Sci.

[33] Kierdorf U, Kierdorf H (2010). Deer Antlers - A Model of Mammalian Appendage Regeneration: An Extensive Review.. Gerontology.

[34] Rolf HJ, Kierdorf U, Kierdorf H, Schulz J, Seymour N (2008). Localization and characterization of STRO-1+ cells in the deer pedicle and regenerating antler.. PLoSOne.

[35] Goss RJ (1983). Deer antlers: Regeneration, function, and evolution.

[36] Riekstina U, Cakstina I, Parfejevs V, Hoogduijn M, Jankovskis G (2009). Embryonic stem cell marker expression pattern in human mesenchymal stem cells derived from bone marrow, adipose tissue, heart and dermis.. Stem Cell Rev.

[37] Liedtke S, Stephan M, Kogler G (2008). Oct4 expression revisited: potential pitfalls for data misinterpretation in stem cell research.. BiolChem.

[38] Panagopoulos I, Moller E, Collin A, Mertens F (2008). The POU5F1P1 pseudogene encodes a putative protein similar to POU5F1 isoform 1.. OncolRep.

[39] Lee J, Kim HK, Rho JY, Han YM, Kim J (2006). The human OCT-4 isoforms differ in their ability to confer self-renewal.. J Biol Chem.

[40] Rustom A, Saffrich R, Markovic I, Walther P, Gerdes HH (2004). Nanotubular highways for intercellular organelle transport.. Science.

[41] Domhan S, Ma L, Tai A, Anaya Z, Beheshti A (2011). Intercellular communication by exchange of cytoplasmic material via tunneling nano-tube like structures in primary human renal epithelial cells.. PLoS One.

[42] Onfelt B, Nedvetzki S, Benninger RK, Purbhoo MA, Sowinski S (2006). Structurally distinct membrane nanotubes between human macrophages support long-distance vesicular traffic or surfing of bacteria.. J Immunol.

[43] Gerdes HH, Bukoreshtliev NV, Barroso JF (2007). Tunneling nanotubes: a new route for the exchange of components between animal cells.. FEBS Lett.

[44] Gerdes HH (2009). Prions tunnel between cells.. Nat Cell Biol.

[45] Sowinski S, Jolly C, Berninghausen O, Purbhoo MA, Chauveau A (2008). Membrane nanotubes physically connect T cells over long distances presenting a novel route for HIV-1 transmission.. NatCell Biol.

[46] Gurke S, Barroso JF, Hodneland E, Bukoreshtliev NV, Schlicker O (2008). Tunneling nanotube (TNT)-like structures facilitate a constitutive, actomyosin-dependent exchange of endocytic organelles between normal rat kidney cells.. Exp Cell Res.

[47] Veranic P, Lokar M, Schutz GJ, Weghuber J, Wieser S (2008). Different types of cell-to-cell connections mediated by nanotubular structures.. BiophysJ.

[48] Brambrink T, Foreman R, Welstead GG, Lengner CJ, Wernig M (2008). Sequential expression of pluripotency markers during direct reprogramming of mouse somatic cells.. Cell Stem Cell.

[49] Ho R, Chronis C, Plath K (2011). Mechanistic insights into reprogramming to induced pluripotency.. J Cell Physiol.

[50] Maherali N, Ahfeldt T, Rigamonti A, Utikal J, Cowan C (2008). A high-efficiency system for the generation and study of human induced pluripotent stem cells.. Cell Stem Cell.

[51] Simons M, Raposo G (2009). Exosomes–vesicular carriers for intercellular communication.. Curr Opin Cell Biol.

[52] Koga K, Matsumoto K, Akiyoshi T, Kubo M, Yamanaka N (2005). Purification, characterization and biological significance of tumor-derived exosomes.. Anticancer Res.

[53] Caby MP, Lankar D, Vincendeau-Scherrer C, Raposo G, Bonnerot C (2005). Exosomal-like vesicles are present in human blood plasma.. Int Immunol.

